# Epidemiological study on calf diarrhea and coccidiosis in dairy farms in Bahir Dar, North West Ethiopia

**DOI:** 10.1186/s13620-020-00168-w

**Published:** 2020-07-21

**Authors:** Habtamu Tamrat, Negesse Mekonnen, Yeshwas Ferede, Rudi Cassini, Negus Belayneh

**Affiliations:** 1 Bahir Dar University, School of Animal Science and Veterinary Medicine, P.O.Box:5501, Bahir Dar, Ethiopia; 2 Department of Animal Medicine, Production and Health, University of Padova, VialeDell’ Universita, 16 Legnaro, 35020 Padova, Italy; 3 Srinka Agricultural Research Centre, P.O. Box 74, Sirinka, Ethiopia

**Keywords:** Coccidiosis, Diarrhea, Dairy calf, Ethiopia, Incidence rate, Longitudinal study, Risk factors

## Abstract

**Background:**

A longitudinal prospective study was conducted from October 2017 to April 2018 on calf diarrhea and coccidiosis in dairy farms in Bahir Dar, North West Ethiopia with the objectives of determining the incidence of calf diarrhea and calf coccidiosis from diarrheic calves, assessing the major risk factors associated with calf diarrhea and coccidiosis and identifying the existing *Eimeria* species. A total of 237 calves, 86 calves from 52 smallholder dairy farms and 151 calves from 8 large dairy farms, were used for this study. Fresh fecal samples were collected from 86 diarrheic calves for identification of *Eimeria* species.

**Results:**

Overall incidences of calf diarrhea and coccidiosis found in this study were 33.5 and 20.1%, respectively. In total, 19 potential risk factors were investigated for their association with calf diarrhea and coccidiosis from diarrheic calves using Cox regression. Age of calf (HR = 2.057, *P* = .002), body condition (HR = 1.802, *P* < .001), house condition (HR = 2.072, *P* = .004) and age at first colostrum feeding time (HR = 2.107, *P* = .002) were found significantly (*P* < 0.05) associated with the risk of diarrhea by multivariate Cox regression. Among the risk factors tested, age (HR = 13.36, *P* < .001) and sex of calves (HR = 3.500, *P* = .020) were found significantly (*P* < 0.05) associated with coccidiosis by multivariate Cox regression. A total of nine *Eimeria* species were identified. *E. bovis* (28.6%), *E. zuernii* (19.0%) and *E. auburnensis* (14.3%) were the most common *Eimeria* species encountered.

**Conclusion:**

The incidence of calf diarrhea and coccidiosis was high in the dairy herds in North West Ethiopia. Therefore, sound dairy calf management practices are needed to mitigate risk factors for calf diarrhea and coccidiosis with a view to reducing the incidence of calf diarrhea and coccidiosis in Ethiopian dairy farms.

## Introduction

Ethiopia possesses the largest livestock population in Africa, which is currently estimated to be 59.5 million cattle including 9.6% under 6 months of age and 8.4% between 6 month and 1 year [1]. In Ethiopia, livestock is a significant contributor to economic and social development at the household and national level. Livestock accounts for 15–17% of total GDP and 35–49% of agricultural GDP and directly contributes to the livelihoods of more than 70% of Ethiopians [2].

Despite the largest livestock population in Ethiopia, livestock diseases are the major constraints to productivity and production causing economic losses to dairy producers in Ethiopia [3, 4]. Among the diseases, neonatal diarrhea of calves is a severe form of diarrhea that causes huge economic loss to dairy producers [5, 6]. Several infectious agents like viruses, bacteria and protozoan parasites cause calf diarrhea [7–9].

*Eimeria* species are the most important protozoan parasites causing calf coccidiosis and affecting calves all over the world and are usually most common and important in calves younger than 1 year old [9, 10]. The occurrence of diarrhea depends upon the interaction of *Eimeria* species, density of *Eimeria* oocysts in the environment, rate of exposure of calves to oocysts, environmental temperature, humidity, sunlight and stressors of the calves. Many coccidia organisms in the environment possibly cause coccidiosis [11]. However, *E. zuernii* and *E. bovis* are the two most common *Eimeria* species which cause coccidiosis in calves 6 to 12 months of age. The result is a marked reduction in feed efficiency, weight loss and diarrhea. This delays heifer age at first calving reducing dairy industry profits [12–16].

Calves are primarily infected through the ingestion of sporulated oocysts and infection can rapidly spread from calf to calf when animals are communally housed and/or overcrowded, and from cow to calf via dirty and contaminated udders [17, 18]. In Ethiopia, *Eimeria* are among the most common diarrhea-causing protozoan enteropathogens in calves and causes severe calf morbidity and mortality [19, 20]. In Ethiopia, though diarrhea is an important cause of calf morbidity and mortality, studies done to quantify the magnitude of the problem and determine the underlying causes are scanty.

Although quite a lot of similar studies on calf coccidiosis have been conducted to determine the prevalence and associated risk of calf coccidiosis in different areas of Ethiopia, it is worth noting that Ethiopia is a large country with a huge amount of livestock populations, mostly cattle, and therefore most of the studies are targeting only specific areas and not the whole country. Unlike other studies this is a longitudinal prospective study and not a cross-sectional one, as all other studies. Longitudinal study design is a far better design in epidemiological studies to determine the incidence of diseases within observational time periods. Therefore, this study was initiated to determine incidence rate of calf diarrhea and coccidiosis in the study area, to identify the existing *Eimeria* species causing calf diarrhea and to investigate major risk factors associated with calf diarrhea and coccidiosis.

## Materials and methods

### Study area

The study was conducted in and around Bahir Dar town, the capital city of Amhara National Regional State. The city is located approximately 565 km Northwest of Addis Ababa, having a latitude and longitude of 11°36′ N and 37°23′ E. The average annual rainfall ranges from 1200 to 1600 mm and temperature 8–31 °C. The altitude of the area ranges between 1500 m–2300 m above sea level. This area has a total population of 345,610, out of which 297,794 are urban inhabitants and the rest are living at rural areas around Bahir Dar [1]. In these areas, smallholder-farming households produce milk mostly from indigenous cattle breeds. Average milk production per cow in the area is about one liter per day, resulting in an estimated milk production of 46,710,335 l per lactation from all lactating cows [21]. The predominant production system in the region is mixed crop-livestock farming and cattle are the most important livestock species reared in this region. Crossbred dairying is being promoted by the regional government through distribution of pregnant crossbred heifers and use of artificial insemination due to the high milk demand and supply variation in the nearby urban and peri-urban centers [22].

### Study farms

There were few relatively large dairy farms with herd size greater than 20 cows and many smallholder dairy farms with herd size less than 20 cows in and around Bahir Dar. For this particular study, a total of 118 study farms were used. In agreement with a study conducted on incidence of calf morbidity and mortality in smallholder dairy farms in Kenya by [23], a smallholder dairy farm was defined as one with at least 1 and at most 20 cattle of all ages and sexes. Dairy producers who had more than 20 dairy cattle during sampling were categorized as large sized dairy farms. Thus, 110 smallholder dairy farms and 8 relatively large dairy farms were considered during this study**.** Local (Fogera) and cross breed (Holstein with Fogera) dairy calves of both sexes reared under smallholder and commercial dairy farms aged from birth up to 1 year were the study population.

### Study design

The study was a longitudinal prospective observational study that extended for 6 months from October 2017 to March 2018 to determine the incidence rate of calf diarrhea and coccidiosis and to investigate their determinant factors. Questionnaires were administered to 110 smallholder and 8 relatively large dairy farm producers. Respondents were briefed on the objective of the study. Herd-level data were taken by in-person interview aimed at soliciting information pertaining to farm characteristics. Accordingly, calf management practices (colostrum management, feeding, housing and health management), information about the age of calves, sex, breed, and usual problems observed in calves and the associated signs were collected for risk assessment. The hygienic status of calf pens and the calves themselves were assessed based on housing system (ventilation, types of barn), bedding, types of floor and body condition of the calves [24] and was categorized as poor or good [25].

### Sampling procedure and sample size determination

Preliminary data were sourced from the Agricultural Office and from the two dairy cooperatives present in the area to obtain the lists of smallholder and commercial dairy producers and to estimate the size of study populations. All calves from the 8 large size dairy farms and a representative random sample of calves from smallholders were selected for the study. Considering individual smallholder dairy farms as a cluster, a cluster-sampling methodology was used to select calves from smallholder dairy farms. A total of 110 smallholder dairy producers were registered in the two dairy cooperatives. Accordingly, 52 smallholder dairy farms were sampled by using a systematic random sampling technique from the sampling frame.

The sample size calculation used in this study was calculated according to [26] using an expected prevalence of calf coccidiosis of 19.0% [27], a 95% confidence interval and a required absolute precision of 5%. In total 237 calves, 86 calves from 52 smallholder dairy farms, and 151 calves from 8 large dairy farms were used for this study. All calves below 1 year at the start of and born during the study period were included in the research. When a selected smallholder farm did not have calves during the 6 month cohort period, another dairy farm mostly from the nearby area replaced it.

### Data collection

Monitoring of selected dairy farms for calf diarrhea and coccidiosis was conducted for 6 months (October 2017 to March 2018). All calves in the selected farms were individually identified and monitored throughout the follow up period. The calves were withdrawn from the follow up when they completed their cohort period or due to sale or deaths. For each calf, individual data, incidents of observed diarrhea and incidence of coccidiosis from diarrheic calves during the monitoring were recorded. Calves were regularly visited every week. Finally, 30 g of fresh fecal samples were collect per rectum from each diarrheic calf using disposable plastic gloves. During sampling, farm type, date of sampling, consistency of the diarrhea (watery or bloody), age, sex, breed, and tag number were recorded for each calf on a recording format. The sample was placed in a labeled clean universal bottle using 2.5% (w/v) potassium dichromate solution and transported in a cool box to the Bahir Dar University, veterinary parasitology laboratory on the same day of collection, and preserved at refrigeration temperature until processing within 48 h of arrival.

### Coproscopical examination

Qualitative fecal examination was conducted using flotation technique for the detection of the oocysts of *Eimeria* using concentrated sucrose solution (Sheather’s sucrose solution) with specific gravity of 1.27 as described by [28]. The slides were examined using a compound microscope (10x objectives). For identification of *Eimeria* species, positive samples were taken to the National Animal Health Diagnostic and Investigation Center, Addis Ababa, Ethiopia. Identification of *Eimeria* oocysts was based on the morphological features of the oocysts (size of oocyst and sporocysts, shape, color, and texture of oocyst wall, presence or absence of micropyle, polar cap) with the aid of taxonomic keys [29–31]. Oocysts size was measured under ocular eye piece that is calibrated with a micrometer under a 40× objective of a microscope. Quantitative fecal examination was performed in positive samples by McMaster technique to determine the number of oocysts of *Eimeria* per gram of feces (OPG), as described by [28, 32].

### Data management and analysis

#### Estimation of incidence rates for diarrhea and Coccidiosis

Diarrhea and coccidiosis are the outcome measures of interest in this study. As animals in this longitudinal study were recruited at different times and were followed for different periods of time, incident density (true rate) was used in describing disease occurrences. The speed at which an event occurs per unit time at risk (true rate) was calculated to define the risk of specific disease conditions. Therefore, the incidence rates of events were estimated by the following formula [23, 33, 34].

$$ \mathbf{IR}=\frac{\mathrm{Number}\ \mathrm{of}\ \mathrm{interest}\ \mathrm{events}\ \mathrm{occurring}\ \mathrm{during}\ \mathrm{observational}\ \mathrm{period}}{\mathrm{Total}\ \mathrm{calf}\hbox{-} \mathrm{days}\ \mathrm{at}\ \mathrm{risk}} $$

The numerator is the number of occurrences of the outcome of interest, whereas the denominator is the number of calf-days at risk from enrollment in the study to the end of the observation period, due to removal from the herd by sale or death or completing observation time in the study period.

In the calculation to describe incidence rate of diarrhea, a calf recovered from diarrhea was considered to be at risk for another diarrheic case after complete recovery (complete disappearance of clinical signs) from the preceding one. The days in which the calf stayed diarrheic were not counted as days at risk [33, 34]. For this study purpose, total calf-days at risk were converted to calf-months at risk. Moreover, to facilitate result comparisons with other findings, and because directly taking true rate results tends to overestimate the event [34, 35], true rates calculated for the event were converted into risk rates based on the formula (Risk rate = 1-e ^-True Rate^) [36].

#### Risk factors analysis

A total of 19 hypothesized explanatory variables for calf diarrhea and coccidiosis were initially considered for analysis. The responses of all variables were dichotomized to facilitate analysis and interpretation of results. Statistical Package for the Social Sciences (SPSS) statistical software version 20 was used to run Cox regression, a statistical method used to model survival analysis. Statistical analyses of the associations between explanatory variables (risk factors) and status variables (outcome variables) were done by Cox’s proportional hazard model. Initially, the association of individual risk factor with an outcome variable was screened by univariate Cox regression. Those variables significantly associated with the outcome variable at 5% or *P* < 0.05 significance level in the univariate analysis were recruited for multivariate analysis using multiple Cox regression to see their independent effect. In the multivariate analysis, a model was fitted for each outcome variable by stepwise backward elimination of insignificant variables (*P* > 0.05). Microsoft Excel 2016 was used to conduct descriptive analyses.

## Results

A total of 237 calves (198 crossbreed and 39 local; 128 males and 109 females) were monitored for 6 months to determine the incidence of calf diarrhea and coccidiosis from the 118 selected dairy farms (110 small holder and 8 relatively large). They contributed 38,023 calf-days at risk, which is also equivalent to 211 calf-months at risk. Out of 237 enrolled calves, 30 (12.7%) withdrew from the study before the termination of the cohort period due to deaths (*n* = 21) and sales (*n* = 9), and they contributed only for the days from enrollment up to death/sale. Overall 86 cases of diarrheic calves were recorded, and 42 were due to coccidiosis. Consequently, the present longitudinal prospective study revealed that calf-months at risk of diarrhea and coccidiosis were 33.5 and 20.1%, respectively (Table 1).

**Table 1:** Incidence (true rate and risk rate) of diarrhea and coccidiosis in Bahir Dar dairy farms

Events	N	Calf- days at risk	Calf-months at risk	Incidence Rate (IR)	
				True rate	Risk rate (%)^a^
Diarrhea	86	38,023	211	0.408	33.5
Coccidiosis	42	33,611	187	0.225	20.1

^a^Risk rates estimated from true rates derived by the formula, Risk rate = 1- e^-true rate^ [36]

### Risk factor analysis

In total 19 explanatory variables were tested separately using univariate Cox regressions. Of these, seven potential risk factors were significantly (*P* < 0.05) associated with risk of calf diarrhea. These included, age of calf, body condition, house hygiene, house condition, age at first colostrum feeding, mother instinct and parity of dam (Table 2).

**Table 2:** Explanatory variables significantly associated with the incidence of calf diarrhea based on univariate and multivariate analysis of cox proportional hazards regression model

Variables	Categories	Univarate Analysis			Multivariate Analyses		
		HR^a^	95% CI	*P* value	HR^a^	95% CI	P value
Age	< 6 months	0.455	0.275–0.754	.002	2.057	1.290–3.279	.002
	6–12 months						
Body Condition	Good	0.253	0.151–0.423	.000	1.802	1.338–2.426	.000
	Poor						
House hygiene	Poor	1.966	1.202–3.215	.007	–	–	–
	Good						
House condition	Separated	0.480	0.301–0.764	.002	2.072	1.253–3.425	.004
	Unseparated						
Parity of dam	Multiparous	0.643	0.416–0.996	.046	–	–	–
	Primiparous						
Age at 1stCF	< 6 h	0.467	0.295–0.740	.001	2.107	1.304–3.404	.002
	> 6 h						
Mother instinct	Good	0.539	0.346–0.841	.006	–	–	–
	Poor						

^a^Hazard ratio (which has similar meaning to relative risk), Age at 1^st^ CF = age at first colostrum feeding

These potential risk factors were further evaluated by a multivariate Cox regression model and age of calves, body condition, housing condition and age at first colostrum feeding were found significantly associated with calf diarrhea in the final model (Table 2).

Regarding calf coccidiosis, out of the total of 19 variables tested, seven potential risk factors were found significantly (*P* < 0.05) associated with the risk of coccidiosis (Table 3) at univariate Cox regression.

**Table 3:** Explanatory variables significantly associated with the incidence of calf coccidiosis based on univariate and multivariate analysis of cox proportional hazards regression model

Variables	Categories	Univarate Analysis			Multivariate Analysis		
		HR^a^	95% CI	*P* value	HR^a^	95% CI	*P* value
Age	< 6 months	13.044	4.627–36.770	.000	13.36	4.622–38.621	.000
	6–12 months						
Sex	Male	6.593	2.580–16.848	.000	3.500	1.213–10.096	.020
	Female						
Breed	Cross	3.591	1.101–11.644	.033	–	–	–
	Local						
Body Condition	Good	0.092	0.022–0.385	.001	–	–	–
	Poor						
House hygiene	Poor	16.157	2.222–117.483	.006	–	–	–
	Good						
House condition	Separated	0.122	0.037–0.398	.000	–	–	–
	Unseparated						
Parity of dam	Multiparous	0.371	0.202–0.684	.001	–	–	–
	Primiparous						

^a^Hazard ratio (which has similar meaning to relative risk)

**Fig. 1:**
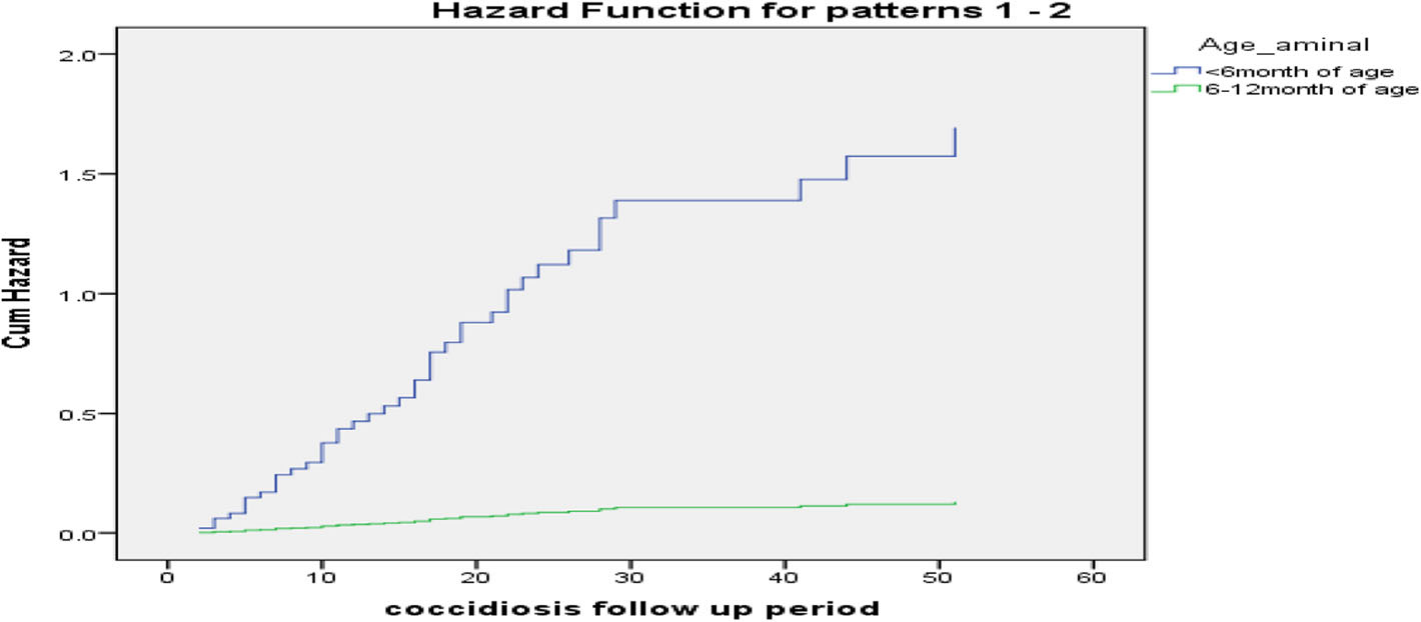
The hazard difference of calf coccidiosis among age group

The multivariate model identified age and sex of calf as significantly associated with calf coccidiosis in the final model. The hazard for calf coccidiosis in male calves was 3.5 times higher than female calves and in younger calves was about 13 times higher than older calves (Table 3 and Fig. 1).

### *Eimeria* species identification

During this study period, all 86 diarrheic calves were sampled according to the established protocol and 42 individuals tested positive for coccidiosis. For each sample the coprocolture and identification of species was performed. A total of nine *Eimeria* species were identified. *E. bovis* and *E. zuernii* were the more prevalent species and accounted for 28.6 and 19.0% of the positive samples, respectively. Other species found were *E. auburnensis, E. canadensis*, *E*. *ellipsoidalis*, *E. subspherica*, *E. cylindrica*, *E. alabamensis* and *E. brasilensis*, with prevalence of 14.3, 11.9, 9.5, 7.1, 4.8, 2.4 and 2.4% respectively. Quantitative fecal examination was performed by McMaster technique to determine the number of oocysts per gram of feces (OPG). The mean, maximum and standard deviation of OPG value were 3307.4, 9400 and 3078.4 respectively.

## Discussion

In the present study, the incidence rate of calf diarrhea was 33.5% which is lower than (42.9%) a study done in Debre Zeit [33] but higher than (25.2%) reported in Bahir Dar milk-shed [34]. The variation of these findings may be due to different study design, different target animal or seasonal variation. However, diarrhea was confirmed as an important disease complex that affects calf health as previously reported in Ethiopia and elsewhere [37, 38].

In this study, age was one of the risk factors affecting calf diarrhea. This finding is in agreement with the reports in Ethiopia [33, 34] both with highest incidence rate of diarrhea in younger calves more than older calves. The finding of this result is also consistent with the results of a study done in Southern Germany [9] where calves under 3 months of age were affected by diarrhea more than older calves. In addition, based on the present study, even if farmers had knowledge on the use of colostrum for calf health, only a very small percentage (17.86%) were aware of the optimal time for colostrum feeding and this could have greatly contributed to the high incidence of calf diarrhea. This finding is consistent with the work done in Debre Zeit by [33] and in the central highland of Ethiopia by [39] indicating a high percentage of failure of passive transfer of immunity in market oriented smallholder dairy farms.

Incidence rate and overall prevalence of coccidiosis from diarrheic calves under the age of 12 months were 20.1 and 48.8% respectively. The overall prevalence of coccidiosis from the present study was in line with a study done on coccidial infection in dairy cattle in Shanghai, China by [40] but other studies showed both higher [25, 41] and lower values [9, 42]. This variation is most likely attributed to the differences in study design, and husbandry practices of the study animals in different countries [43] but the results of the present study confirmed the importance of coccidiosis among the causes of calf diarrhea in Ethiopia.

In the present investigation, there was a significant (*P* < 0.05) association of age of calf with the risk of coccidiosis infection. This finding is in agreement with other reports in Ethiopia [25] and outside [9]. Younger calves < 6 months of age were at higher risk of coccidiosis infection than older calves.

Additionally, there was also a statistically significant association (P < 0.05) between sex and coccidiosis infection, with a higher incidence of coccidiosis in male calves than female calves.

This finding is in line with the report by [25] but disagrees with other research where sex has no effect on coccidiosis [9, 42] and with other studies done on adult cattle, which reported higher incidence of coccidiosis in female animals than in male cattle [44, 45]. Nevertheless, this could be attributed to the physiological stress loaded on adult female animals in relation to pregnancies and giving birth as compared to males [42]. The higher incidence in male calves of our study could be due to less care given to male calves as compared to the female calves that are deemed future cows.

In the present study, positive samples from diarrheic calves were further investigated for coccidiosis based on morphological features of different *Eimeria* oocysts. Worldwide, more than 13 *Eimeria* species are reported in cattle. However, in the present study, nine *Eimeria* species were isolated, with *E. bovis* (28.6%) followed by *E. zurnii* (19.0%) and *E. auburnensis* (14.3%) as the most prevalent species. The present finding is in line with other previous reports that suggested *E. bovis* as the most frequently reported *Eimeria* species [17, 25, 46, 47]. In a study conducted in Ethiopia by [25] *E. wyomingensis* and *E. bukidnonensis* were isolated but these species were not in the present study. This could be attributed to variation in agro-ecology and climate preference of the parasite. The number of *Eimeria* species in mixed infections per examined diarrheic sample ranges from 2 to 5 which is consistent with other studies [25, 30, 48, 49].

The present study revealed that mean, maximum and standard deviation of OPG values were 3307, 9400 and 3078.4 respectively. This finding is similar to what reported by other studies in Ethiopia [25] but higher to values considered normal in healthy animals according to international standards [30, 49, 50]. However, the impact on the health of the animals depends also on the pathogenic potential of the involved *Eimeria* species. The finding that *E. bovis* and *E. zuernii* (two of the most pathogenic species in cattle) were the most common ones among the nine *Eimeria* species identified, suggest that some animals of the study population is probably harbouring high burden of dangerous species. Anyhow, this aspect needs more in-depth investigations.

## Conclusion

The present study revealed that there was a higher incidence of calf diarrhea and coccidiosis in the study area. A number of potential risk factors were tested to determine significant association with incidence of diarrhea and coccidiosis. Age of calves, body condition, house condition and age at first colostrum feeding were significantly associated with the risk of diarrhea while age and sex of the calves were the only factors associated with the risk of coccidiosis. The average level of oocysts per gram of faeces and the widespread presence of two highly pathogenic species (*E. bovis* and *E. zuernii)* is particularly alarming. Based on the finding of this study, due attention should be given to the timing of colostrum feeding in calves and dairy farmers should implement better calf management practices.

## Data Availability

Not applicable.

## Abbreviations

ADG: Average Daily Gain
DHM: Dairy Herd Management
GDP: Gross Domestic Product
ILCA: International Lactation Consultation Association
OPG: Oocyst Per gram of faeces
Rpm: Revolution per Minute
SPSS: Statistical Packages for Social Science
W/V: Weight in Volume

## References

[1] Central Statistical Agency (2016). The Federal Democratic Republic of Ethiopia Agricultural Sample Enumeration Statistical Abstract.

[2] Central Statistical Authority (2011). Agricultural sample survey 2010–2011. Report on livestock and livestock characteristics II. Addis Ababa, Ethiopia. Statistical Bulletin No. 505.

[3] Sissay M, Uggla A, Waller PJ (2008). Prevalence and seasonal incidence of larval and adult cestode infections of sheep and goats in Eastern Ethiopia. Trop Anim.

[4] Sintayehu Y, Fekadu B, Azage T, Berhanu G (2008). Dairy production, processing and marketing systems of Shashemene-Dilla area, South Ethiopia. Improving productivity and market success of Ethiopian farmers project working paper 9.

[5] Millemann Y (2009). Diagnosis of neonatal calf diarrhea. Revue Méd Vét.

[6] Yamamoto T, Nakazawa M (1997). Detection and sequences of the entero aggregative *Escherichia coli* heat stable enterotoxin gene in ETEC strains isolated from piglets and calves with diarrhea. J Clin Microbial.

[7] Razzaque MA, Al-Mutawa T, Mohammed SA (2010). Diarrhea in Pre-Weaned Calves: Relative Risk Rates for Morbidity and Mortality in 13 Commercial Farms of Hot Arid Zone. Am J Anim Vet Sci.

[8] Abdullah M, Akter R, Kabir S, Khan M, Abdulaziz M (2013). Characterization of bacterial pathogens isolated from calf diarrhoea in Panchagarh district of Bangladesh. J Agric Food Tech.

[9] Gillhuber J, Rügamer D, Pfister K, Scheuerle M (2014). Giardiosis and other entero pathogenic infections: a study on diarrhoeic calves in Southern Germany. BMC Res Notes.

[10] Yousef D, Yagoob G, Freydoun N, Zahra E, Saied S (2011). Study on prevalence rate of Coccidiosis in diarrheic calves in East-Azerbaijan province. AdvaEnvirons Biol.

[11] Daugschies A, Najdrowski M (2005). Eimeriosis in cattle: current understanding. J Vet Med B Infect Dis Vet Public Health.

[12] DHM (1998). Coccidiosis: the silent thief, dairy herd management magazine. De Graf WDC, VAnopdenbosch, E., Ortega-mora, L.M.

[13] Dedrickson BJ. Coccidiosis in beef calves. Feed Lot Magazine Online, 10:1. Available athttp://www.feedlotmagazine.com.html. 2000.

[14] Kennedy JM (2001). Coccidiosis in cattle. AGRIFACTS.

[15] Kirkpatrick S (2003). Coccidiosis in cattle. Oklahom states university extension facts, available on the Worldwide Web’s at http/agewebwkstates.edu/p.

[16] Pilarczyk B, Balicka-Ramisz A (2004). Occurrence of protozoa Eimeria and Cryptosporidium in calves from west pomerania. Acta Sci Pol Zootechnica.

[17] Faber JE, Kollmann D, Heise A, Bauer C, Failing K, Burger HJ (2002). *Eimeria* infection in cows and their calves: oocyst extraction and levels of specific serum and colustrum antibodies. Vet Parasitol.

[18] Nasir A, Avais M, Khan MS, Ahmad N (2008). Prevalence of Cryptosporidium parvum infection in Lahore (Pakistan) and its association with diarrhea in dairy calves. Int J Agric Biol.

[19] Tadele K, Hailu D, Girma K (2014). Prevalence of calves coccidiosis in Jimma town dairy farms, South-Western Ethiopia, Jimma University Colleges of Agriculture and Veterinary Medicine.

[20] Eyuel T (2016). Occurrence of Coccidiosis in diarrheic calves in and around Asella town dairy farms.

[21] Asaminew T (2007). Production, handling, traditional processing practices and quality of milk in Bahir Dar milkshed area, Ethiopia.

[22] Department of Agriculture (2000). Annual progress reports. Bahir Dar Zuria woreda, Ethiopia.

[23] Muraguri GR, McLeod A, McDermott JJ, Taylor N (2005). The incidence of calf morbidity and mortality due to vector-borne infections in smallholder dairy farms in Kwale District, Kenya. Veterinary Parasitology.

[24] Curt A (2005). Senior extension associate Department of Agricultural and Biological Engineering, pro-dairy program, Cornell University. Dairy Calves and Heifers: Integrating Biology and Management Conference.

[25] Abebe R, Wossene A, Kumssa B (2008). Epidemiology of Eimeria infections in calves in Addis Ababa and Debre Zeit dairy farms, Ethiopia. Intern J Appl Res Vet Med.

[26] Thrusfield M. Veterinary epidemiology. 3rd ed: Blackwell Science Ltd. Royal (Dick) School of Veterinary Studies, University of Edinburgh; 2007.

[27] Tadesse Y, Awol M, Zemzem M (2016). Prevalence and risk factor identification of calf Coccidiosis in and around Bahir Dar town in Amhara regional state, north West Ethiopia, Wollo University, School of Veterinary Medicine, journal of animal research.

[28] Hendrix C (1998). Diagnostic Veterinary Kashmir Valley. Global Veterinarian.

[29] Soulsby EJL (1982). Helminthes, Arthropods, and Protozoas of Domestic Animals.

[30] Kennedy MJ, Kralka RA (1987). A survey of Eimeria species in cattle in Central Alberta. Can Vet J.

[31] Sommer C (1998). Quantitative characterization, classification and reconstruction of oocyst shapes of Eimeria species from cattle. Parasitology..

[32] Kaufmann J. Parasitic infections of domestic animals. Diagnostic Manual. 1996:8–10.

[33] Wudu T (2004). Calf morbidity and mortality in dairy farms in Debre Zeit and its envirions, Ethiopia. MSc thesis.

[34] Yeshwas F. Epidemiological determinants and magnitude of calf morbidity and mortality in Bahir Dar milk-shed, north West Ethiopia MSc thesis: Addis Ababa University, College of Veterinary Medicine and Agriculture, Department of Clinical studies; 2015.

[35] Gitau GK, Perry BD, McDermott JJ (1994). The incidence, calf morbidity and mortality due to Theileria parva infections in smallholder dairy farms in Murang ‘a,District, Kenya. Prev Vet Med.

[36] Martin SW, Meek AH, Willeberg P (1987). Veterinary Epidemiology: Principle and Methods.

[37] Olsson SO, Viring S, Emanuelsson U, Jacobsson SO (1993). Calf diseases and mortality in Swedish dairy herds. Acta Vet Scand.

[38] Sivula NG, Ames TR, Marsh WE (1996). Management practices and risk factors for morbidity and mortality in Minnesota dairy heifer calves. Prev Vet Med.

[39] Amoki OT (2001). Management of dairy calves in Holleta area, central highlands of Ethiopia. Faculty of veterinary medicine.

[40] Hui D, Zhao Q, Han H, Jiang L, Zhu S, Li T (2012). Prevalence of coccidial infection in dairy cattle in shanghai, China. J Parasitol.

[41] Matjila P, Penzhorn B (2002). Occurrence and diversity of bovine coccidia at three localities in South Africa. Vet Parasitol.

[42] Nagwa IT, Faragalla ME, Soad EH (2011). Diagnosis of Eimeriosis in cattle by ELISA using partially purified antigen. Worl App Sci J.

[43] Radostits OM, Gay CC, Hinchcliff KW (2007). Veterinary medicine: a textbook of the diseases of cattle, horses, sheep, pigs and goats.

[44] Priti M, Sinha SRP, Sucheta S, Verma SB, Sharma SK, Mandal KG (2008). Prevalence of bovine coccidiosis at patna. J Vet Paristol.

[45] Tauseef UR, Khan MN, Sajid M, Abbas RZ, Arshad M, Iqbal Z (2011). Epidemiology of Eimeria and associated risk factors in cattle of destrict Toba Teksingh, Pakistan. Parasitol Res.

[46] Speer CA, Howard JL, Smith RA (1999). Coccidiosis:current veterinary therapy, food animal practice.

[47] Waruiru R, Kyvsgaard N, Thamsborg S, Nansen P, Bøgh H, Munyua W (2000). The prevalence and intensity of Helminth and Coccidial infections in dairy cattle in Central Kenya. Vet Res Commun.

[48] Ernst JV, Stewart TB, Witlock DR (1987). Quantitative determination of coccidian oocysts in beef calves from the coastal plain area of Georgia (USA). Vet Parasitol.

[49] Arslan M, Tuzer E (1998). Prevalence of bovine eimeridosis in Thracia, Turkey. Turk J Vet Anim Sci.

[50] Munyua W, Ngotho J (1990). Prevalence of Eimeria species in cattle in Kenya. Vet Parasitol.

